# High-Throughput Sequence Analyses of Bacterial Communities and Multi-Mycotoxin Profiling During Processing of Different Formulations of *Kunu*, a Traditional Fermented Beverage

**DOI:** 10.3389/fmicb.2018.03282

**Published:** 2019-01-09

**Authors:** Chibundu N. Ezekiel, Kolawole I. Ayeni, Obinna T. Ezeokoli, Michael Sulyok, Deidre A. B. van Wyk, Oluwawapelumi A. Oyedele, Oluwatosin M. Akinyemi, Ihuoma E. Chibuzor-Onyema, Rasheed A. Adeleke, Cyril C. Nwangburuka, Jana Hajšlová, Christopher T. Elliott, Rudolf Krska

**Affiliations:** ^1^Department of Microbiology, Babcock University, Ilishan Remo, Nigeria; ^2^Center for Analytical Chemistry, Department of Agrobiotechnology (IFA-Tulln), University of Natural Resources and Life Sciences, Vienna (BOKU), Tulln, Austria; ^3^Microbiology and Environmental Biotechnology Research Group, Agricultural Research Council-Institute for Soil, Climate and Water, Pretoria, South Africa; ^4^Unit for Environmental Sciences and Management, North-West University, Potchefstroom, South Africa; ^5^Department of Agriculture and Industrial Technology, Babcock University, Ilishan Remo, Nigeria; ^6^University of Chemistry and Technology, Prague, Czechia; ^7^Institute for Global Food Security, School of Biological Sciences, Queen’s University Belfast, Belfast, United Kingdom

**Keywords:** bacterial diversity, fermented beverage, food safety, high-throughput sequencing, lactic acid bacteria, *kunu*, mycotoxins

## Abstract

*Kunu* is a traditional fermented single or mixed cereals-based beverage popularly consumed in many parts of West Africa. Presently, the bacterial community and mycotoxin contamination profiles during processing of various *kunu* formulations have never been comprehensively studied. This study, therefore, investigated the bacterial community and multi-mycotoxin dynamics during the processing of three *kunu* formulations using high-throughput sequence analysis of partial 16S rRNA gene (hypervariable V3-V4 region) and liquid chromatography tandem mass spectrometry (LC-MS/MS), respectively. A total of 2,303 operational taxonomic units (OTUs) were obtained across six processing stages in all three *kunu* formulations. Principal coordinate analysis biplots of the Bray-Curtis dissimilarity between bacterial communities revealed the combined influences of formulations and processing steps. Taxonomically, OTUs spanned 13 phyla and 486 genera. Firmicutes (phylum) dominated (relative abundance) most of the processing stages, while Proteobacteria dominated the rest of the stages. *Lactobacillus* (genus taxa level) dominated most processing stages and the final product (*kunu*) of two formulations, whereas *Clostridium sensu stricto* (cluster 1) dominated *kunu* of one formulation, constituting a novel observation. We further identified *Acetobacter*, *Propionibacterium*, *Gluconacetobacter*, and *Gluconobacter* previously not associated with *kunu* processing. Shared phylotypes between all communities were dominated by lactic acid bacteria including species of *Lactobacillus*, *Lactococcus*, *Leuconostoc*, *Pediococcus*, and *Weissella*. Other shared phylotypes included notable acetic acid bacteria and potential human enteric pathogens. Ten mycotoxins [3-Nitropropionic acid, aflatoxicol, aflatoxin B_1_ (AFB_1_), AFB_2_, AFM_1_, alternariol (AOH), alternariolmethylether (AME), beauvericin (BEAU), citrinin, and moniliformin] were quantified at varying concentrations in ingredients for *kunu* processing. Except for AOH, AME, and BEAU that were retained at minimal levels of < 2 μg/kg in the final product, most mycotoxins in the ingredients were not detectable after processing. In particular, mycotoxin levels were substantially reduced by fermentation, although simple dilution and sieving also contributed to mycotoxin reduction. This study reinforces the perception of *kunu* as a rich source of bacteria with beneficial attributes to consumer health, and provides in-depth understanding of the microbiology of *kunu* processing, as well as information on mycotoxin contamination and reduction during this process. These findings may aid the development of starter culture technology for safe and quality *kunu* production.

## Introduction

Fermented beverages constitute a major part of the diets of traditional African homes (Tafere, 2015). In Nigeria, traditional beverages are widely consumed and mostly preferred to commercial soft drinks by individuals from low income settings due to their relatively low cost of production and high nutritional benefits (Ezekiel et al., 2015).

*Kunu* is a traditional beverage produced principally from single or mixed cereals such as maize, millet, rice, or sorghum. In some cases, peanut is added to the cereals to make-up the raw material input. The grain (cereals and nuts) input could also be supplemented with additives such as cloves, pepper, ginger, sweet potato and tiger nut, which are added as homogenized mixtures just before the fermentation of the beverage. The sets of cereal and nut applied to the production of *kunu* determine its variety. For example, *kunu-zaki* comprises of millet, sorghum or maize; *kunu-tsamiya*, millet, sorghum or rice; *kunu-gyada*, rice, peanut, millet or sorghum; and *kunu-gayamba*, solely millet (Gaffa et al., 2002). *Kunu-zaki* is the commonest of the *kunu* varieties due to its nutritional and health benefits (Adelekan et al., 2013). Generally, *kunu* is consumed whilst in an active state of fermentation by both adults and children (Efiuvwevwere and Akona, 1995). The nutritional content of *kunu* includes 9.84–12% carbohydrate, 1.56–3% protein, 0.1–0.3% fat, and 0.61–075% dietary minerals (Adeyemi and Umar, 1994; Badifu et al., 1999), and its health benefits range from purging the bowels and relief of flatulent conditions (Omakwu, 1980), to the enhancement of lactation in nursing mothers (Efiuvwevwere and Akona, 1995).

The production process of *kunu* comprises six critical steps: steeping of the cereals in water to allow for softening and fermentation by autochthonous bacteria, wet milling, gelatinization of a large portion of milled grains by addition of boiling water, addition of a mix of milled additives and the remainder portion of the milled grains to the gelatinized gruel, fermentation of the mixture, and sieving of the fermented slurry to obtain *kunu* (Gaffa and Ayo, 2002). The pH of *kunu* is usually acidic (pH 3–5.46) (Efiuvwevwere and Akona, 1995; Gaffa et al., 2002; Adelekan et al., 2013). The steeping duration varies and is largely dependent on the type of cereal used (Gaffa et al., 2002). Similarly, the duration of the fermentation step of the gelatinized gruel mix varies from 8 to 24 h depending on the complexity of the food matrices used for *kunu* processing and the proportion of mixture of milled grains and additives to the gelatinized gruel (Gaffa and Ayo, 2002; Osuntogun and Aboaba, 2004; Oluwajoba et al., 2013; Olosunde et al., 2015).

The fermentation stages of *kunu-zaki* are driven by consortia of bacteria (mostly lactic acid bacteria) (Efiuvwevwere and Akona, 1995; Gaffa and Gaffa, 2004; Osuntogun and Aboaba, 2004; Oguntoyinbo et al., 2011; Ikpoh et al., 2013; Aboh and Oladosu, 2014) and a few yeasts (notably *Saccharomyces cerevisiae*) (Efiuvwevwere and Akona, 1995; Gaffa and Gaffa, 2004) that contribute to the breakdown of complex macromolecules into simpler compounds. Although, there is a recent culture-independent (sanger-based sequencing technology) microbiological study of a *kunu* variety (Oguntoyinbo et al., 2011), most of the previous microbiological studies on *kunu* are based on conventional isolation methods and classical identification techniques which are prone to biases, low taxonomic resolution, misidentification of species and underestimation of species richness and diversity (Cocolin and Ercolini, 2015; Ezeokoli et al., 2016). Currently, there are no high-throughput sequencing (HTS)-based studies on *kunu* microbial ecology. The application of HTS technologies (also referred to as next generation sequencing) may help unravel hitherto unidentified bacterial species associated with *kunu* processing and *kunu* products (Franzosa et al., 2015; Ezeokoli et al., 2018). In addition, there is a paucity of information on the microbial diversity of different formulations of *kunu* at different stages of processing. Consequently, an in-depth understanding of the microbiology of *kunu* will provide insight into the community structure and functional roles of microbes in the production of varieties of this beverage. Furthermore, the knowledge of *kunu* microbial community will facilitate the selection of starter cultures for improvement of the safety and quality of this widely consumed beverage.

Chemical food contaminants (e.g., mycotoxins) may, however, distort the safety and quality of *kunu* due to the use of diverse cereals and nuts that have been reported to be prone to several mycotoxins in the beverage formulation (Adetunji et al., 2014; Afolabi et al., 2015; Ezekiel et al., 2015, 2018; Oyedele et al., 2017). Thus, there is a need to evaluate the extent to which mycotoxins can be carried over into various *kunu* formulations, considering the diverse grain inputs into the production of this beverage. Previous studies have shown that a few mycotoxins can be present in *kunu-zaki*, albeit at reduced levels (Ezekiel et al., 2015; Olosunde et al., 2015). However, the influence of specific processing steps on the levels of mycotoxins during the processing of one or many formulations of *kunu* is yet to be elucidated. Such data coupled with information on the microbial community structure is highly relevant to food safety and protection of consumer health in view of the adverse health effects that may arise from dietary mycotoxin exposures (International Agency for Research on Cancer [IARC], 2015). Furthermore, it has been postulated that traditional beverages can contribute to increased mycotoxin exposures in high cereal -dependent regions such as sub-Saharan Africa (SSA) (Ezekiel et al., 2018). Thus, there is a need to determine the processing steps and grain combinations that are critical to the reduction of mycotoxin exposure through beverage consumption. This information may be useful for recommending safe *kunu* formulation(s) for consumer benefits.

Therefore, this study aimed to determine the bacterial community diversity and dynamics during the processing of three *kunu* formulations by using HTS-based technology, and to evaluate the effect of processing on the mycotoxin profiles at various stages of processing by using liquid chromatography tandem mass spectrometry (LC-MS/MS).

## Materials and Methods

### Source of Ingredients

Samples of millet, sorghum (red and white varieties), ginger, peanut, sweet potato, tiger nut and cloves were purchased in January 2017 from the local market in Ilishan Remo (6.8932°N, 3.7105°E), Ogun state, Nigeria. Only ingredients without visible insect infestation, discolorations and rot were used for this study.

### *Kunu* Formulations and Processing

Three different formulations of *kunu* designated as A, B, and C were prepared using the ingredients. Formulation A comprised of millet, white sorghum, peanut, cloves, ginger and tiger nut while formulation B was made from millet, white sorghum, cloves, ginger and sweet potato. Formulation C consisted of millet, red sorghum, cloves, ginger and tiger nut. In the formulations, cloves, ginger, sweet potato and tiger nut served as additives to millet, sorghum (white or red variety) and peanut, which were used as grain bases for the beverage. Precisely 1 kg of each grain and 100 g of each additive were used in the formulations. Maize was excluded from the formulations in order to eliminate extremely high levels of diverse mycotoxins from the beverage.

**FIGURE 1:**
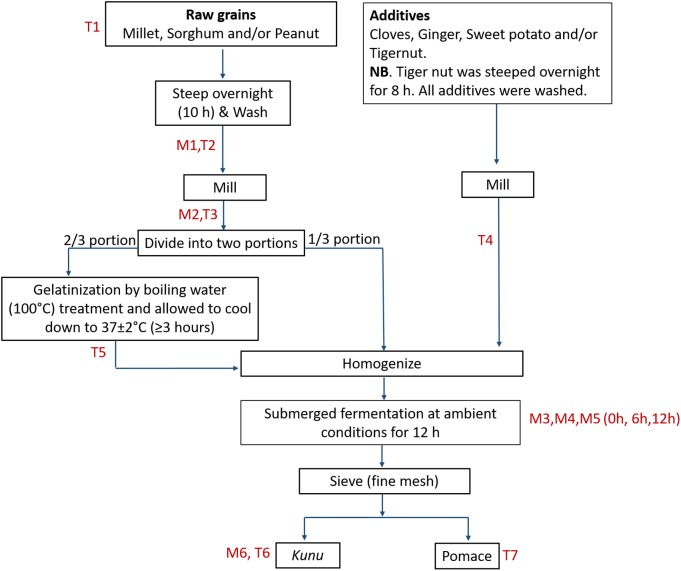
Flow for *kunu* processing. M and T represent points of sample collection for bacterial community and mycotoxin analyses, respectively.

For *kunu* processing, rudimentary utensils and tap water were used in order to replicate as much as possible the traditional processing method. The exact process undertaken for the preparation of the *kunu* formulations in this study is outlined in Figure 1. For clarity, all processing steps, including the fermentation step, were carried out under prevailing ambient temperature (33 ± 2°C). Precisely 3 L and 300 mL of tap water were used for the steeping of grains (1 kg) and additives (100 g), respectively; this step and the fermentation step were performed in prewashed 5 L wide mouth plastic containers with lids and without agitation or additional aeration. Prewashing of the containers was performed with detergent and thorough rinsing with tap water. Ginger and sweet potato were not steeped, but peeled and washed with tap water prior to wet milling. Milling (wet milling) was performed for approximately 10 min using a commercial milling machine to make fine slurry. The commercial milling machine was rinsed twice with tap water to clear off debris from previous use. Gelatinized portions of the gruels were left to cool down to a temperature of 37 ± 2°C (typically for duration of at least 3 h) before proceeding to the homogenization step. Sieving of the fermented substrate was performed using a clean muslin cloth.

### Sampling

Samples were collected from various core processing steps based on relevance of each step to either the bacterial community profile study or mycotoxin reduction analysis. For both (bacterial and mycotoxin) analyses, samples of steep liquor, milled grains and *kunu* were collected, while samples of raw grains, milled additives, cooled gruel (after gelatinization) and pomace (after sieving) were taken for mycotoxin analysis, and samples of the fermenting substrate were collected at 0, 6, and 12 h of fermentation for microbial analysis. Approximately 20 mL and 20 g subsamples of liquid and semi-/solid samples, respectively, were randomly collected for analysis. All samples were immediately frozen at -20°C prior to shipment on dry ice and analyses at the Agricultural Research Council–Biotechnology Platform, South Africa for HTS analysis, and the Center for Analytical Chemistry, IFA–Tulln, Austria for multi-mycotoxin analysis.

### Total Community DNA Extraction

Genomic DNA was extracted from samples using the Quick-DNA Fecal/Soil microbe kit (Zymo Research, Irvine, CA, United States) according to the manufacturer protocol. For steep liquor, 20 ml of samples was vacuum filtered through a sterile 0.2 μm pore size membrane filter (Whatman Plc, Maidstone, United Kingdom) and DNA extracted directly from the membrane filter whereas for milled grains and fermenting gruels, 0.25 g (wet weight) of sample homogenate were used for DNA extraction. For *kunu*, samples were centrifuged at 12,000 rpm for 5 min and DNA was extracted from 0.25 g (wet weight) of the sediment. DNA integrity and concentration were verified by agarose (1% w/v) gel electrophoresis and fluorometric quantification (Qubit 2.0, Invitrogen, Carlsbad, CA, United States), respectively.

### High-Throughput Sequencing of Bacterial Communities

Partial 16S rRNA gene (hypervariable V3-V4) libraries were amplified using universal bacterial primers 341F (forward) and 805R (reverse) (Klindworth et al., 2013). Each forward and reverse primer contained Illumina overhang adapters (Illumina Inc., United States). Library preparation steps were performed as described previously (Mashiane et al., 2017; van Wyk et al., 2017). Briefly, PCR amplicons were purified using Agent Court AMPure XP beads (Beckman Coulter, Brea, CA, United States), and each sample amplicon uniquely indexed with dual indexes. Uniquely indexed amplicons were purified again with AMPure beads, quantified using a Qubit fluorometer (Invitrogen, Carlsbad, CA, United States), normalized to equal concentration in a resuspension buffer (Illumina Inc., San Diego, CA, United States) library, pooled in equimolar proportions, denatured in 0.2 M NaOH and loaded along with denatured PhiX (control) library onto a MiSeq V3 cartridge for a 2 X 300 bp paired-end sequencing run on the Illumina MiSeq sequencer (Illumina Inc, San Diego, CA, United States).

### Bioinformatics

Demultiplexed sequence reads were checked for quality using FastQC software (v. 0.11.7, Babraham Institute, United Kingdom) and subsequently trimmed of low-quality regions (10 bp) at both 5′- and 3′-ends using Trimommatic (Bolger et al., 2014). PANDAseq (Masella et al., 2012) was used to assemble (merge) forward and reverse reads with a minimum overlap of 50 bp, as well as eliminate merged reads with ambiguous nucleotide bases (N) and spurious lengths (>465 bp) at a threshold (t) of 0.7 and by using the simple Bayesian algorithm. Merged reads were then binned (closed reference OTU picking) into operational taxonomic units (OTUs) (97% 16S rRNA gene similarity) against the SILVA rRNA reference (Release 128) (Quast et al., 2013) by using Usearch61 (Edgar, 2010; Edgar et al., 2011) in Quantitative Insights Into Microbial Ecology (QIIME) software (v. 1.9.1) (Caporaso et al., 2010). Singletons were removed from the OTU count table before normalization (rarefaction) to even depths across samples in QIIME. Alpha diversity and principal coordinate analyses were performed in QIIME and/or R software version 3.4.0 (R Core Team, 2013). Multivariate analysis was performed based on the relative abundance of OTU counts. Statistical tests for differences in multivariate space were not performed because treatments were not replicated.

### Data Availability

Raw sequence reads generated in this study are available in the Sequence Read Archives^1^ of the National Centre for Biotechnology Information under the bioproject accession number PRJNA482055.

### Multi-Microbial Metabolite Analysis of *Kunu* Formulations

Samples of grains, by-products (steep liquor and pomace) and *kunu* were analyzed for the presence of over 295 microbial metabolites including the major mycotoxins (e.g., aflatoxins, fumonisins, dexoynivalenol, ochratoxins, and their metabolites) by a liquid chromatography tandem mass spectrometric (LC-MS/MS) method described by Malachova et al. (2014). Please see full list of 295 metabolites in Malachova et al. (2014). For the grains, 5 g of each ground sample was extracted with 20 mL of acetonitrile/water/acetic acid (79:20:1, v/v/v) in a 50 mL polypropylene tube (Sarstedt, Nümbrecht, Germany) for 90 min using a GFL 3017 rotary shaker (GFL 3017, Burgwedel, Germany). In the case of liquid samples (steep liquor and *kunu*), mycotoxins were extracted from 2.5 mL of the samples in 15 mL polypropylene tubes containing 7.5 mL of extraction solvent and centrifuged at 10,000 rpm for 3 min at ambient temperature. All extracts were diluted with acetonitrile/water/acetic acid (20:79:1, v/v/v) solvent and injected into the LC system as described in detail by Sulyok et al. (2006). LC-MS/MS screening of target fungal metabolites was performed using a QTrap 5500 LC-MS/MS System (Applied Biosystems, Foster City, CA, United States) equipped with TurboionSpray electrospray ionization source and a 1290 Series HPLC System (Agilent, Waldbronn, Germany). Chromatographic separation was performed at 25°C on a Gemini^®^C18-column, 150 × 4.6 mm i.d., 5 mm particle size, equipped with a C18 4 × 3 mm i.d. security guard cartridge (Phenomenex, Torrance, CA, United States). Confirmation of positive analyte identification was obtained by the acquisition of two scheduled multiple reaction monitoring (MRMs) which yielded 4.0 identification points according to the European Commission decision 2002/657. In addition, the LC retention time and the intensity ratio of the two MRM transitions agreed with the related values of an authentic standard within 0.1 min and 30% rel., respectively. Apparent recoveries of the metabolites were determined by spiking 0.25 mL of five different *kunu* samples. The spiked samples were stored overnight at ambient temperature to establish equilibrium between the metabolites and samples. The extraction (in 1 mL of solvent), dilution and analysis were as described earlier. The accuracy of the method was crosschecked by participation in inter-laboratory comparison studies organized by BIPEA (Gennevilliers, France). Only mycotoxins that were positive in the samples are reported in the results section.

## Results

### Diversity and Community Structure of Operational Taxonomic Units

**FIGURE 2:**
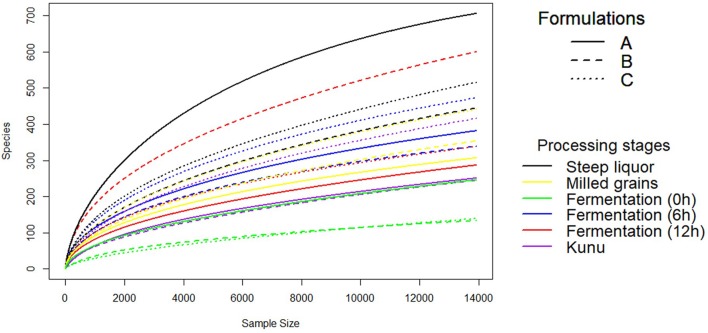
Rarefaction Curve of OTUs to even subsampling depth. Rarefaction curve was constructed using the vegan package of R software after single rarefaction to a depth of 13920 sequences per sample in QIIME software.

**FIGURE 3:**
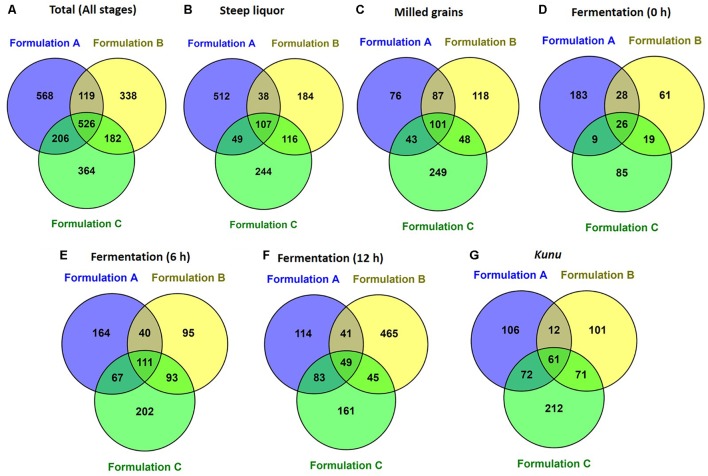
Shared operational taxonomic units between formulations **(A)** Total (all stages) **(B)** Steep liquor. **(C)** Milled grains. **(D)** Fermentation at 0 h. **(E)** Fermentation 6 h. **(F)** Fermentation 12 h. **(G)** *Kunu*. Venn diagram was constructed by using the online Venny tool v.2.1. (Oliveros, 2007; http://bioinfogp.cnb.csic.es/tools/venny/).

A total of 2,647,697 high-quality sequences were obtained from all the samples of the three *kunu* formulations after quality filtering and binning into OTUs. Normalization (rarefaction) of data to a depth of 13,920 sequences per sample was sufficient to estimate community diversity in all samples (Figure 2). A total of 2,303 OTUs were obtained from all samples (Figure 3A), with 526 OTUs shared between all formulations (Figure 3A). Steep liquor had the highest number of OTUs in both formulations A (706 OTUs) and C (516 OTUs), while fermentation at 12 h had the highest number of OTUs (600 OTUs) in formulation B (Table 1). As expected, trends in Chao1—, a species richness estimation that accounts for possible rare species in the community that might have been missed due to under sampling—, phylogenetic diversity—an indices based on evolutionary distances between species in a given sample—, and Shannon-Weiner index of diversity observed among stages of the different formulations were similar to trends observed in the number of OTUs (Table 1). In comparison to stages within a formulation and between formulations, higher values of these indices indicate higher species richness (Observed OTUs, Chao1) and diversity (Shannon–Weiner index, Phylogenetic diversity). In all formulations, the least OTU diversity was observed at the 0 h fermentation stage. Similarly, the least number of shared OTUs among all the formulations was at the 0 h fermentation, while the most number of shared OTUs was mid-way through the fermentation (6 h) (Figures 3B–G). Overall, pair-wise comparisons of shared OTUs between formulations revealed that the most number of OTUs (206 OTUs) were shared between formulation A and C (Figure 3A), while the least number of OTUs (119 OTUs) were shared between formulations A and B (Figure 3A).

**Table 1:** Operationaltaxonomic units (OTUs) diversity metrics in different formulations of *kunu* after single rarefaction to even depth of 13,920 sequences per sample.

	Processing stage	Observed OTUs	Chao1	^†^Phylogenetic diversity	Shannon-Weiner
Formulation A	Steep liquor	706	811.99	49.40	6.21
	Milled grains	307	515.41	17.81	4.42
	Fermentation (0 h)	246	448.78	18.32	2.99
	Fermentation (6 h)	382	575.80	21.12	4.85
	Fermentation (12 h)	287	521.50	19.03	3.97
	*Kunu* (final product)	251	493.00	19.96	1.77
Formulation B	Steep liquor	445	720.34	27.14	5.15
	Milled grains	354	665.73	21.42	3.95
	Fermentation (0 h)	134	249.56	10.72	1.50
	Fermentation (6 h)	339	505.96	20.33	4.45
	Fermentation (12 h)	600	918.31	33.64	6.23
	*Kunu* (final product)	245	477.23	17.09	2.86
Formulation C	Steep liquor	516	874.50	29.79	4.65
	Milled grains	441	738.01	27.55	4.56
	Fermentation (0 h)	139	293.29	15.13	1.70
	Fermentation (6 h)	473	738.42	25.03	5.19
	Fermentation (12 h)	338	582.17	20.18	4.53
	*Kunu* (final product)	416	818.21	26.69	3.96


*^†^Based on PD_whole_tree; Observed OTUs or OTU Richness is the number of observed unique operational taxonomic units (defined by 97% 16S rRNA gene sequence similarity). Chao1 is a richness estimation or prediction based on the adjustment of species richness for rare OTUs that may have been missed due to under sampling. Chao 1 is often referred to as the true species richness of a given community; PD (phylogenetic distance) whole tree is a diversity estimate calculated from the phylogenetic distances of OTUs present within a sample. Shannon-Weiner index of diversity accounts for the abundance and evenness (equitability) of species in a community. The higher the Shannon-Weiner index, the more diverse are the species in that community.*

**FIGURE 4:**
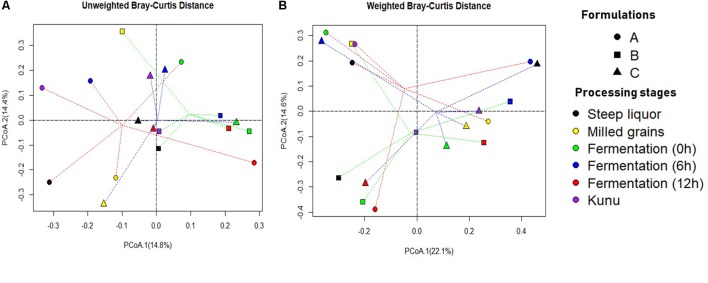
Principal coordinate analysis (PCoA) Biplot of Bray-Curtis Dissimilarity between operational taxonomic units of different *kunu* formulations. **(A)** Unweighted (presence/absence of OTUs). **(B)** Weighted (presence/absence and relative abundance of OTUs). Dotted lines red, green and blue show distance of every sample to formulation A, formulation B, and formulation C group centroid, respectively. PCoA was constructed using the ‘ape’ package of R software. Bray-Curtis dissimilarity is a statistic used to estimate differences in the composition of species between two or more sites/samples based on counts.

The unweighted (on the basis of presence/absence of OTUs) Bray-Curtis dissimilarity principal coordinate analysis (PCoA) biplot for OTUs distribution revealed that the bacterial community structure of the processing stages of formulation A were the most diverse compared to other formulations (Figure 4A). However, close similarities in community structure were observed between stages of the same formulations and between stages of different formulations (Figures 4A,B). For example, the bacterial community of fermentation at 0, 6, and 12 h were similar for formulation B (Figure 4A), while the bacterial community of the fermenting substrate at 0 h was similar for both formulations B and C (Figure 4A). Similarly, the bacterial community of steep liquor and *kunu* for formulation B were similar, while the bacterial community of the fermenting substrate at 12 h and *kunu* of formulation B were closely similar. In the weighted (on the basis of presence/absence and relative abundance of OTUs) Bray-Curtis dissimilarity PCoA biplot for the absence/presence and relative abundance of OTUs (Figure 4B), the similarities between communities were more pronounced, particularly for communities between *kunu* of formulation A and milled grains of formulation B (Figure 4B). Within formulations, steep liquor, fermentation at 0 h and *kunu* had closely similar bacterial community structures (Figure 4B). Other closely similar bacterial communities included those of steep liquor in formulation C and fermentation at 6 h in formulation A.

### Taxonomic Diversity and Dynamics

**FIGURE 5:**
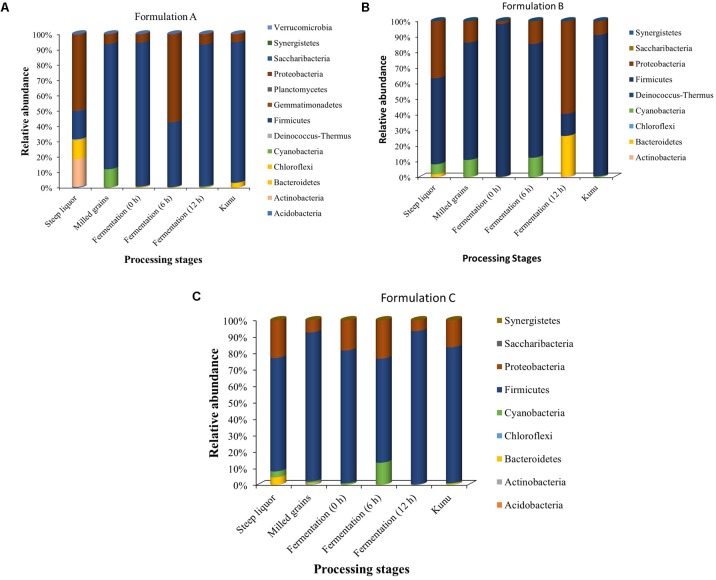
Relative abundance of classifiable OTUs at the phylum taxonomic rank. **(A)** Formulation A **(B)** Formulation B **(C)** Formulation C. OTUs not assigned to a phylum taxonomic rank are excluded from the bar plot.

All 2,303 OTUs obtained from the processing stages of the different *kunu* formulations taxonomically spanned at least 13 phyla and 486 genera, with several of the OTUs being unclassified at these taxonomic ranks. At the phylum taxonomic level, Firmicutes dominated most of the processing stages of all *kunu* formulations (Figure 5), except for the steeping stages of formulation A, and at 6 and 12 h fermentation stages of formulations A and B, respectively, which were dominated by Proteobacteria (Figure 5). Other phyla that constituted at least 1% relative abundance in all stages included Actinobacteria, Bacteriodetes, and Cyanobacteria (Figure 5).

**FIGURE 6:**
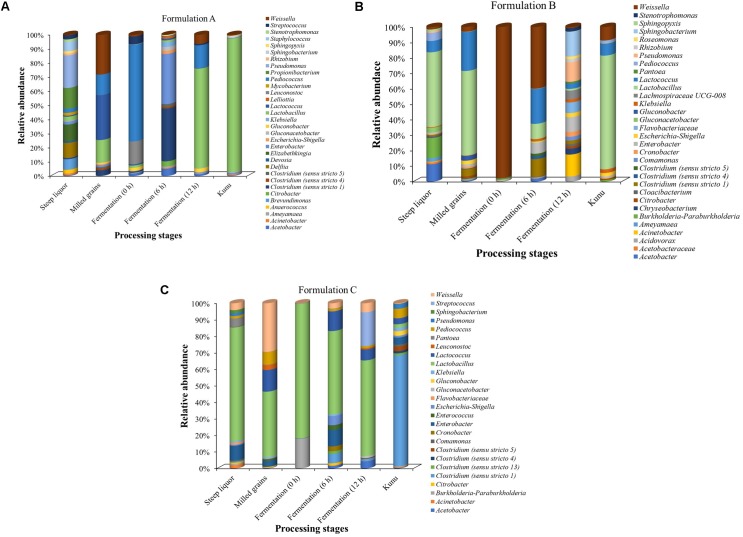
Relative abundance of classifiable OTUs with at least 1% relative abundance at the genus taxa level. Family names are shown where OTUs are unclassifiable at the genus level. **(A)** Formulation A. **(B)** Formulation B. **(C)** Formulation C. OTUs not classifiable to genus taxonomic rank were excluded from the bar plot.

The phylotypes (at the genus taxonomic rank) with at least 1% relative abundance in any of the processing stages of each formulation are presented in Figure 6. Overall, *Lactobacillus* dominated most of the processing stages, especially in the *kunu* of formulations A and B (Figures 6A,B). In contrast, *Lactobacillus* dominated all stages but *kunu* of formulation C, where *Clostridium sensu stricto* (cluster 1) was found to dominate *kunu* of formulations C (Figure 6C). In formulation A, *Acetobacter*, *Brevundimonas*, *Clostridium*, *Delftia*, *Devosia*, *Elizabethkingia*, *Enterobacter*, *Lactobacillus*, *Lactococcus*, *Leuconostoc*, *Pediococcus*, *Propionibacterium*, *Pseudomonas*, *Staphylococcus*, *Streptococcus*, *and Weissella* each constituted at least 5% relative abundance in at least one processing stage (Figure 6A). In formulation B, *Lactobacillus*, *Lactococcus*, *Acetobacter*, *Burkholderia*/*Paraburkholderia*, *Weissella*, *Enterobacter*, *Clostridium sensu stricto* (cluster 1), *Sphingobacterium*, *Acinetobacter*, and *Pseudomonas* each constituted at least 5% relative abundance in at least one processing stage (Figure 6B). In formulation C, *Burkholderia/Paraburkholderia*, *Clostridium sensu stricto* (cluster 1), *Enterobacter*, *Lactobacillus*, *Lactococcus*, *Pediococcus*, *Streptococcus*, *and Weissella* each constituted at least 5% relative abundance in at least one processing stage (Figure 6C). Across formulations, some genera which constituted at least 1% (but less than 5%) relative abundance (Figures 6A–C) and whose species have potential roles in food fermentations include *Gluconobacter* and *Gluconacetobacter*.

Bacterial succession pattern was observed at the fermentation stages of all the *kunu* formulations. For formulation A, *Pediococcus* (67.5%) dominated the initial fermentation (0 h) (Figure 6A), while *Clostridium* (35.6%) and *Enterobacter* (33.8%) dominated at 6 h and *Lactobacillus* (68.9%) at 12 h fermentation. In contrast, *Weissella* dominated the initial fermentation (97.3%) and mid-stream (6 h) fermentation (32.9%) in formulation B while *Acinetobacter* (12.8%), *Sphingobacterium* (14.6%), *Pseudomonas* (11.5%) and *Enterobacter* (8.7%) were the relatively more abundant phylotypes in the final fermentation (12 h) stage of the same formulation (Figure 6B). On the other hand, in formulation C, *Lactobacillus* spp. dominated all the three stages of fermentation (Figure 6C).

**FIGURE 7:**
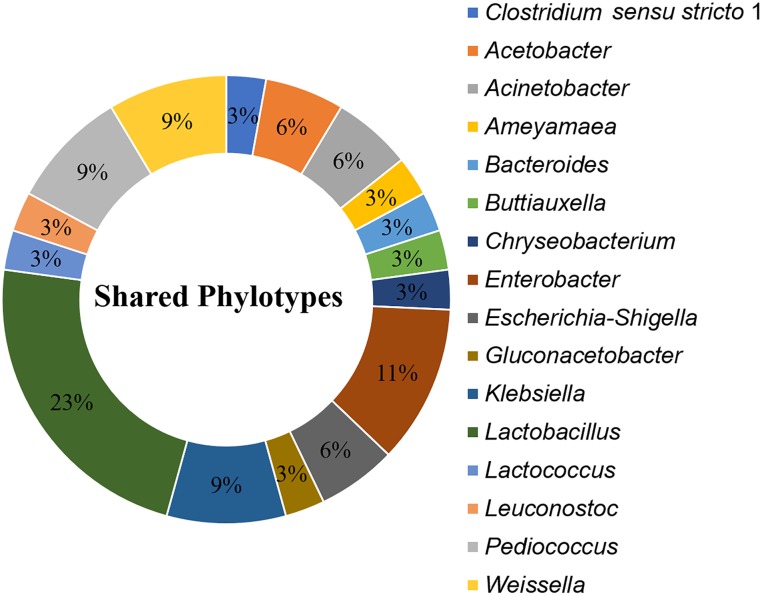
Shared phylotypes (genus taxa level) between all processing stages of *kunu* formulations. OTUs unclassified at genus taxa level are excluded from the plot. Pie slices indicate relative abundance of individual phylotypes in the dataset. Shared phylotypes were computed using the “shared_phylotypes.py script in QIIME software.

A total of 35 OTUs, comprising 16 phylotypes (at the genus taxonomic rank) were common to all processing stages and formulations (Figure 7). These “shared phylotypes” were dominated by *Lactobacillus* spp. (17%). Other shared phylotypes included species of *Enterobacter, Gluconacetobacter Lactobacillus*, *Lactococcus*, *Leuconostoc*, *Pediococcus*, and *Weissella*.

### Multiple Mycotoxins in *Kunu* Formulations

#### Occurrence of Mycotoxins in the Ingredients (Grains and Additives)

A total of 10 mycotoxins were found in the ingredients used for processing the three *kunu* formulations (Table 2). Aflatoxins were found in almost all grains (except the white sorghum variety) and additive. Only the B-aflatoxins were detected in the grains. The mean levels of aflatoxin B_1_ (AFB_1_) were higher in red sorghum (113 μg/kg), peanut (51.5 μg/kg), and tiger nut (26.1 μg/kg) compared to millet (0.67 μg/kg), while almost similar mean concentrations of AFB_2_, 6.56 and 9.27 μg/kg, were quantified in peanut and red sorghum, respectively. AFM_1_ was detected in peanut and red sorghum at mean concentrations of 1.7 and 4.22 μg/kg, respectively, while citrinin (CIT) was only quantified in millet at mean concentration of 5.64 μg/kg. The concentrations of aflatoxicol, alternariol (AOH), alternariolmethylether (AME), and beauvericin (BEAU) in grains did not exceed 5 μg/kg. In contrast, the concentrations of 3-Nitropropionic acid (3-NPA) were very high in peanut (1121 μg/kg) and red sorghum (936 μg/kg). Moniliformin (MON) levels were higher in millet (68.7 μg/kg) and white sorghum (44.2 μg/kg) than in red sorghum (2.93 μg/kg) and peanut (1.42 μg/kg).

**Table 2:** Mycotoxin levels in grains and nuts for *kunu* formulation.

Mycotoxins	Limit of detection (μg/kg)	Mycotoxin concentrations (μg/kg) ± standard deviation^1^				
		
		Peanut	Millet	White sorghum	Red sorghum	Tiger nut
3-Nitropropionic acid	0.8	nd	4.15 ± 0.61	0.28 ± 0.01	nd	0.25 ± 0.02
Aflatoxin B_1_	0.24	1121 ± 231	10.8 ± 0.01	nd	936 ± 514	9.97 ± 0.50
Aflatoxin B_2_	0.4	3.69 ± 1.52	nd	nd	4.12 ± 1.81	1.68 ± 0.02
Aflatoxin M_1_	0.4	51.5 ± 20.1	0.67 ± 0.01	nd	113 ± 34.2	26.1 ± 0.40
Aflatoxicol	1	6.56 ± 0.93	nd	nd	9.27 ± 1.20	2.50 ± 0.01
Alternariol	0.4	nd	1.89 ± 0.61	nd	nd	5.64 ± 0.40
Alternariolmethylether	0.032	1.42 ± 1.19	44.2 ± 20.4	68.7 ± 13.7	2.93 ± 0.28	5.06 ± 0.20
Beauvericin	0.008	3.35 ± 0.07	nd	0.61 ± 0.04	nd	1.73 ± 0.20
Citrinin	0.16	1.71 ± 1.41	nd	nd	4.22 ± 2.11	0.82 ± 0.00
Moniliformin	1.6	3.60 ± 0.11	nd	nd	nd	1.51 ± 0.10


*^*1*^Standard deviation from the mean (number of samples = 3). nd, not detected.*

### Changes in Mycotoxin Levels During Processing of the Three *Kunu* Formulations

**FIGURE 8:**
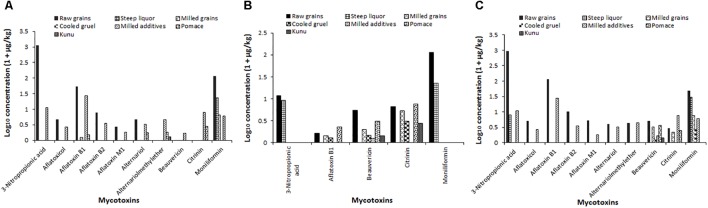
Changes in mycotoxin levels during the processing of three *kunu* formulations (**A**: *kunu* made from millet, white sorghum, peanut, cloves, ginger, and tiger nut; **B**: *kunu* from millet, white sorghum, cloves, ginger, and sweet potato; **C**: *kunu* made from millet, red sorghum, cloves, ginger, and tiger nut).

The distribution of mycotoxin concentration data were normalized by logarithmic (Log_10_) transformation. The value “1” was first added to the mycotoxin concentration in a sample, and then the resulting value was transformed to give the data reported in Figure 8. Estimates of percentage reduction of mycotoxins due to processing were based on percentage differences between mycotoxin levels in the raw grains and processing steps and *kunu* (Okeke et al., 2015). The changes in mycotoxin concentrations during processing of *kunu* formulation A is shown in Figure 8A. Steeping and milling of the raw grains caused a reduction below the detectable limits of 3-NPA, AFB_2_, AFM_1_, aflatoxicol, AOH, AME, BEAU and CIT levels in the milled grains, as well as 99.5% and 95.2% reduction of AFB_1_ and MON contents of the raw grains from 52.2 and 114 μg/kg to 0.25 and 5.53 μg/kg, respectively, in the milled grains (Figure 8A). The residual AFB_1_ and MON levels in the milled grains were further reduced to undetectable levels in the cooled gruel. The milled additives re-introduced all the 10 mycotoxins into the beverage process system at varying levels (Figure 8A). However, the fermentation stage caused complete reduction of 3-NPA, aflatoxicol, AFB_2_, AFM_1_, BEAU and MON to undetectable levels in the *kunu* product. Although AFB_1_, CIT and AOH were also not found in *kunu* of formulation A, about 2, 26, and 32% of the respective 26.5, 6.79, and 2.26 μg/kg in the milled additives were residual in the pomace (a bye-product of *kunu* processing; please see Figure 1). Only 9% of 3.5 μg/kg AME in the milled additives was carried over into the *kunu* (Figure 8A).

Only five of the 10 mycotoxins present in the ingredients were detected during the processing of formulation B *kunu* (Figure 8B). 3-NPA, AFB_1_ and MON were not detected in the *kunu* despite their occurrence at different processing steps. For 3-NPA and MON, at least 75% of the levels in the raw grains (10.8 and 106 μg/kg, respectively) were quantified in the steep liquor. About 9% (0.43 μg/kg) of the BEAU in the ingredients (4.97 μg/kg) and 40% of CIT in the milled grains (4.32 μg/kg) were carried over into *kunu* (Figure 8B). During the processing of *kunu* formulation C (Figure 8C), steeping and milling of the raw grains as well as the fermentation stage resulted in reduction of the concentrations of 3-NPA, AFB_1_, AFB_2_, AFM_1_, aflatoxicol, AOH and AME to undetectable levels. In addition, 16% (0.45 μg/kg) of the BEAU in the ingredient (milled grains and additives; 2.91 μg/kg) was carried over to *kunu*, with the sieving step removing the remainder portion of the toxin (Figure 8C). No detectable levels of the 8.7 μg/kg of CIT and 52.3 μg/kg of MON inputted into the processing system by the grains and additives were found in the *kunu*. However, significant reduction of MON and CIT were accomplished by the steeping and fermentation stages, respectively. Sieving also contributed to further reduction of CIT levels (Figure 8C).

## Discussion

This study reports, for the first time, on the application of HTS in the delineation of bacterial community structure during processing of *kunu vis-a-vis* changes in mycotoxin concentrations. Overall, this study clearly indicates the core bacterial communities and succession during the various stages of processing of three formulations of *kunu*, and the possible role of the microbial communities in reducing the mycotoxin levels (from ingredients to the final *kunu* product) irrespective of formulations.

The high number of OTUs (species richness) obtained during the steeping stage of the various *kunu* formulations suggests a rich genetic diversity of bacteria in the grains. Although potable tap water used for the steeping was not evaluated independently for microbial diversity, it is also likely that potable water contributed to the species diversity at the steeping stage. Furthermore, this observation of high species richness may be due to the stepping environment and duration serving as a broth and permitting the proliferation, respectively, of the autochthonous microbial species. Similar observations of diverse bacteria occurring during the steeping of raw grains for *kunu-zaki* production were suggested in previous studies (Efiuvwevwere and Akona, 1995; Gaffa and Gaffa, 2004). The high number of OTUs in formulations A and C, and shared OTUs between these two *kunu* formulations in contrast to the relatively lower number of OTUs obtained for the formulation B could be attributed to the composition of ingredients used for the various formulations: only formulations A and C contained tiger nut. This observation suggests that tiger nut may serve as the source of the additional OTUs in formulations A and C. Tiger nut grows underground and has been reported to harbor a plethora of microbiota consequent on its direct interaction (not enclosed in pods) with the soil environment (Ayeh-Kumi et al., 2014; Gambo and Da’u, 2014; Ike et al., 2017).

Herein, we put forward some hypotheses to explain the observed changes in OTU richness between successive stages of *kunu* processing. The lower OTU richness observed in the milled grains in comparison to OTU richness in steep liquor could be attributed to the fact that during the overnight steeping, bacterial species associated with the grains and steep water proliferate, giving rise to a fermented broth—the steep liquor (usually with bubbles or foams due to CO_2_ release observed). Since this fermented broth (or steep liquor) are decanted and discarded before subsequent washing of the steeped grains, a large number of species present in the steep liquor are not carried over to the milling stage. Furthermore, since the steep liquor is not used in subsequent processing step, the OTUs detected in the milled grains are likely those that are within the grains, OTUs not completely eliminated during washing of the steeped grains, and/or any OTUs introduced by chance contamination during milling of the grains. Hence, the milled grain is expected to contain significantly lower species than the steep liquor as observed in all formulations. Between the milled grains and onset of fermentation, a reduction in OTUs was observed. This relatively low OTU diversity observed at the 0 h fermentation time compared to the pre-gelatinization stage in all the *kunu* formulations is attributable to the hot water (100°C) treatment during the preceding gelatinization of the milled grains. Certainly, such heat treatment will have at least a bacteriostatic (if not significantly bactericidal) effect on most of the indigenous microbiota, consequently contributing to reduced species diversity (Russell, 2003). In a study by Akharaiyi and Omoya (2008), following heat treatment of fermented maize (ogi) at 100°C, the total viable count of microorganisms drastically reduced to minimal levels. Despite expected increase in species after the addition of the un-heated 1/3 portion, the restoration of species richness to the pre-gelatinization state may have not been detected due to dilution effect thereby resulting in a lower chance of species (or their DNA) recovery during DNA extraction. Bias in the efficiency of recovery and detection of rare species is a well-known limitation of metagenomics approaches (Wooley and Ye, 2010; Breitwieser et al., 2017).

During the fermentation stage, an increase in OTUs was observed between the onset and first 6 h of fermentation. *Kunu* fermentation is a lactic acid fermentation type, with initial slightly acidic pH of about 6.0 (Gaffa and Gaffa, 2004), which gradually reduces to an acidic pH of about 4.76 (Gaffa and Gaffa, 2004) at the end of 12 h and then to 3.0 in the *kunu* product (Efiuvwevwere and Akona, 1995; Gaffa et al., 2002; Adelekan et al., 2013). Increase in acidity is a selective pressure on the microbial community during fermentation (Wolfe and Dutton, 2015; Zabat et al., 2018). We hypothesize that the observed increase in OTUs between the first 6 h was due to the moderate pH and other favorable conditions (e.g., temperature, oxygen availability, absence of growth inhibition metabolites) during this period, permitting the growth of species which were suppressed by the heat treatment in the gelatinization stage (Yang et al., 2016). However, as the pH further decreases (selection pressure) toward the end of fermentation (at 12 h), only adapted species (mostly lactic acid bacteria) proliferate (Gaffa and Gaffa, 2004). Hence, a reduction (compared to at 6 h) in OTU diversity at the end of fermentation was observed in formulations A and C. In contrast, an increase in OTU richness was observed between the 6 and 12 h of fermentation in formulation B. The reason for this observation may be due to the presence of sweet potato and the absence of tiger nut in formulation B compared to formulations A and C. Difference in substrate types influences the genetic and functional diversity and dynamics of bacteria during fermentation, and also play a role in the equilibrium of biochemical reactions and conditions (e.g., pH, oxygen availability, redox reactions, enzymatic activities, and temperature) during fermentation (Giraffa, 2004; Ijabadeniyi, 2007; Van der Meulen et al., 2007). Furthermore, the observed general reduction in OTUs after the sieving step may be attributed to the removal of a large number of species in the pomace; the pomace contains fibrous material which provides a larger surface area for the adhesion of microorganisms compared to the liquid *kunu* fraction.

In view of the afore discussions on OTU diversity, the trend in numbers of OTUs obtained from the mid-stream (6 h) fermentation stage up to the final product stage for the formulations suggests the proliferation of adapted (after heat treatment) functional species (key drivers of biochemical conversions of macromolecules in the fermenting substrate) and development of a climax/streamlined bacterial community observed in the *kunu* product. The associations observed between the bacterial community structure (as indicated by the ordination plots) of processing stages and/or different formulations suggest that, indeed, ingredients and processing steps influence microbial community composition and dynamics.

Taxonomically, the Firmicutes was the dominant phylum during most of the *kunu* processing stages and in the final product. Also, similar to observations of several culture-dependent microbiological studies on *kunu* (Efiuvwevwere and Akona, 1995; Gaffa and Gaffa, 2004; Osuntogun and Aboaba, 2004) and a culture-independent study of retailed *kunu* (Oguntoyinbo et al., 2011), lactic acid bacteria (LAB) were generally dominant during various processing stages of the three *kunu* formulations. With this finding, the dominant role of LAB during *kunu* production becomes more obvious and explanations for the acidic pH of *kunu* reported in earlier studies become clearer (Efiuvwevwere and Akona, 1995; Gaffa et al., 2002; Adelekan et al., 2013). In particular, *Lactobacillus* spp. was the most dominant of the LAB species in most of the fermentation stages of all three formulations and in *kunu* of most formulations. Similar observation for the dominance of *Lactobacillus* spp. in *kunu-zaki* was reported by Inyang and Dabot (1997) and Oguntoyinbo and Narbad (2012). The high occurrence of *Lactobacillus* in these formulations suggests that *Lactobacillus* spp. play a vital role during *kunu* processing and further adds to the growing scientific evidence on the predominance of this bacteria during processing of cereal-based fermented products such as *ogi* (Oguntoyinbo et al., 2011; Okeke et al., 2015), *boza* (Gotcheva et al., 2000), and *bushera* (Muyanja et al., 2003). In addition, *Lactobacillus* spp. are multifunctionally diverse, including many species with probiotic properties (Sanni et al., 2013; Oh et al., 2018) and physiological capabilities for the breakdown of complex polysaccharides in human and animal diets (Barrangou et al., 2006); thus, they could be a reason for some of the nutritional and health benefits generally associated with *kunu* consumption (Omakwu, 1980; Efiuvwevwere and Akona, 1995).

In contrast to the general assertions that the microbial community of *kunu* is dominated by *Lactobacillus*, *Clostridium sensu stricto* (cluster 1) dominated bacterial community of *kunu* product in formulation C. Although species of *Clostridium* have been previously reported in some *kunu* variety (e.g., *kunu-zaki*) (Oguntoyinbo et al., 2011), this is the first report associating the *Clostridium sensu stricto* (cluster 1) group with *kunu* as well as the dominance of the cluster. Until recently, the phylogenetic differentiation (and hence taxonomic classification) of some species within the *Clostridium sensu stricto* from other *Clostridium* species has been unclear, making their functional role in human diet and health, as well as in food fermentations unclear (Collins et al., 1994; Wiegel et al., 2006; Gupta and Gao, 2009; Kaur et al., 2014). A few species in the *Clostridium sensu stricto* cluster are being reclassified and functionally annotated (Wiegel et al., 2006; Gupta and Gao, 2009; Lopetuso et al., 2013; Yutin and Galperin, 2013). In fact, there is growing scientific evidence for their association with the human gut (Magne et al., 2006; Jacquot et al., 2011) and potential applications as hetero-fermenters in food fermentations and industry (Wiegel et al., 2006). Based on the findings of the study, *kunu* is a suitable source for the targeted isolation of species of the *Clostridium sensu stricto* (cluster 1) group. Subsequently, studies may explore the functional and metabolic traits of these species in *kunu* as well as their suitability as starter cultures for *kunu* fermentation or other cereal-based fermented beverages. Similarly, *Acetobacter*, *Gluconobacter*, *Gluconacetobacter* and *Propionibacterium* genera were present during *kunu* processing, especially in the steeped grains. To the best of our knowledge these species have not been previously reported in the microbiology of *kunu* processing. *Acetobacter*, *Gluconobacter* and *Gluconacetobacter* are acetic acid fermenters that have relevant industrial usefulness due to their ability to convert several sugars and alcohols into industrially important organic acids (e.g., vinegar) (Gupta et al., 2001; Sengun and Karabiyikli, 2011; Mamlouk and Gullo, 2013). *Acetobacter* have been identified in other cereal-based fermented foods such as *burukutu*, a fermented traditional sorghum-based *beer* (Oguntoyinbo, 2014), while *Gluconobacter* and *Gluconacetobacter* are associated with acidic beers (Bokulich and Bamforth, 2013; Mamlouk and Gullo, 2013; Spitaels et al., 2014a). Conversely, *Propionibacterium* is mainly associated with dairy and fermented dairy products (Moslemi et al., 2016), with some species being able to improve the probiotic properties of lactic acid bacteria when incorporated into the vegetables during the production (fermentation) of sauerkraut and other vegetable salads (Babuchowski et al., 1999).

The succession in specific taxa during the processing of *kunu* formulations elucidate functional roles of the dominant species at specific time points as well as their adaptation to the prevailing fermentation conditions. The observation of a streamlined and relatively dominant community of lactic acid bacteria, including species of *Lactobacillus*, *Lactococcus*, *Leuconostoc*, *Pediococcus*, *Weissella* and possibly species within the *Clostridium sensu stricto* clusters, toward the end of fermentation and in the final *kunu* product is related to their adaptation to the acidity pH of *kunu* fermentation which is expected as they are the drivers of the biochemical reactions and generators of lactic acid during the process (Gaffa and Gaffa, 2004; Amadou et al., 2011; Oguntoyinbo et al., 2011; Oguntoyinbo, 2014). As discussed earlier, these species are the drivers of the fermentation and are involved in the metabolic interconversions during fermentation as well as instigators of the selective pressure predisposing succession patterns in species/OTU diversity observed during the fermentation stages of *kunu* processing.

In the present study, several phylotypes common to all processing stages and final product of all formulations were identified. These common (or shared) phylotypes may constitute the core bacterial diversity of *kunu* production. The occurrence of these phylotypes throughout the fermentation stages and among all formulations suggest their key roles in *kunu* fermentation and earmark them as potential starter cultures for *kunu* processing. Some of these shared phylotypes, including species of *Enterobacter*, *Gluconacetobacter*, *Lactobacillus*, *Leuconostoc Pediococcus* and *Weissella*, have been previously reported in *kunu* (Amusa and Odunbaku, 2009; Nwachukwu et al., 2010; Oguntoyinbo et al., 2011), lactic acid-fermented west African cereal-based beverages such as *ogi*, *koko*, and *akasa* (Adebayo-tayo and Onilude, 2008; Oguntoyinbo, 2014; Okeke et al., 2015), cocoa fermentation (Nielsen et al., 2007), Spanish farm cheese (Abriouel et al., 2008) and *suan-tsai* (fermented mustard) from Taiwan (Chao et al., 2009). The role of these core microbiota during the fermentation of *kunu* may include the breakdown complex polysaccharides into simpler useful monomers (e.g., mannitol, a low-calorie sugar) (Hemme and Foucaud-Scheunemann, 2004; Ozogul and Hamed, 2018) and generation of aroma compounds that impact characteristic flavor to fermented food products. Some of these species, for example *Leuconostoc* spp., may also synthesize antibacterial compounds such as bacteriocins (Sawa et al., 2010) that help to eliminate pathogenic bacteria during fermentation or in the fermented food product (Ozogul and Hamed, 2018). The possible production of these useful chemical compounds by these species and other known beneficial/probiotic species found in the *kunu* samples, further underlines the health benefits associated with *kunu* consumption. Similarly, *Enterobacter hormaechei* have been previously reported during processing of fermented products such as lambic beer (Spitaels et al., 2014b) and *inyu*, a fermented black bean sauce (Wei et al., 2013). Also, strains of *E*. *hormaechei* are known to produce food additives and stabilizers such as trehalose (Richards et al., 2002). However, some *E*. *hormaechei* strains can cause infections in humans (Davin-Regli et al., 1997). Certainly, isolation and whole genome sequencing of some *E*. *hormaechei* strains associated with *kunu* processing may help elucidate its direct role (beneficial or pathogenicity) in this traditional beverage. As earlier mentioned, *Gluconacetobacter* is an acetic acid bacterium; however, its role in food fermentation is not yet completely understood. It has been associated with traditional balsamic vinegar (Gullo et al., 2006), however, *Gluconacetobacter is* also regarded as a spoilage organism in acidic beer (Bokulich and Bamforth, 2013; Mamlouk and Gullo, 2013). Nonetheless, the role of species of *Gluconacetobacter* during *kunu* processing could be a possible involvement in the oxidation of ethanol to acetic acid (Gomez-Manzo et al., 2010).

It is obvious from this study that the grains used for producing the various formulations of *kunu* were contaminated with different concentrations of several mycotoxins, albeit at levels that were lower than typical levels in maize batches applied to production of traditional fermented beverages in SSA (Okeke et al., 2015; Ogara et al., 2017; Ezekiel et al., 2018). Mycotoxin contamination of millet, sorghum, peanut and tiger nut have been previously reported in Nigeria (Adebajo, 1993; Makun et al., 2007, 2009; Rubert et al., 2013; Oyedele et al., 2017) and the associated public health concerns with such contaminations are well documented. A worrisome finding is the detection of all 10 mycotoxins in tiger nut; this suggests that tiger nut contributes significantly to the levels of mycotoxins during *kunu* processing. The AFB_1_ level in tiger nut was about 13 times higher than 2 μg/kg; this is of concern because tiger nut is not only applied as an ingredient during *kunu* production but also popularly consumed as snacks (Belewu and Abodunrin, 2006; Gambo and Da’u, 2014). Thus, extracting the juice from this nut before adding it as ingredients during *kunu* processing and discarding the bran (Olaoye et al., 2015) could be explored to lower the mycotoxin contribution from this ingredient. A safer option is to exclude tiger nut from the list of ingredients intended for *kunu* processing and replace it with coconut or bambara nut. This is in view of the frequent feeding of *kunu* to young children as a complementary beverage in some parts of Nigeria (Olaoye et al., 2015).

Aside quantifying the mycotoxin levels in the ingredients, one main objective of the mycotoxin aspect of this study was to determine whether toxins present in the ingredients used for the various *kunu* formulations will be reduced and to what extent in the final product. This point was clearly established in this study as concentrations of all the mycotoxins reported in this study were reduced to non-detectable levels in the *kunu* irrespective of the formulations, except for AME, BEAU and CIT which retained very minimal levels (<2 μg/kg) in the final product of the various formulations. The findings of this study agree with previous reports that suggested that traditional processing significantly reduced the levels of mycotoxins in fermented foods and the extent of reduction depends on the mycotoxin content of grain inputs (Ezekiel et al., 2015; Okeke et al., 2015, 2018). The data on changes in mycotoxin levels during processing of the *kunu* indicated that steeping of the grains contributed the most to reducing several of the mycotoxins. This observation may be substantiated by the diverse phylotypes and high number of OTUs observed at this processing stage. During steeping, diverse bacterial genera were observed including those previously reported to be associated with various mechanisms of mycotoxin reduction (Shetty and Jespersen, 2006; Adebo et al., 2015); although in this study we did not search for the degradation products of the mycotoxins that were reduced. A further reduction of the contents of many of the mycotoxins was observed during the fermentation stage, and microbial data suggest community succession and competitive exclusion of species leading toward the development of a climax community. Lactic acid fermentation has been reported to play a significant role in mycotoxin reduction because the microbiota in the fermenting substrate could either bind mycotoxins to their cell wall (Byun and Yoon, 2003; Oluwafemi and Da-Silva, 2009; Huang et al., 2017) or degrade/biotransform them (Shetty and Jespersen, 2006; Adebo et al., 2015). Aside fermentation, simple dilution that occurs during the gelatinization step and sieving were also useful to reduce the levels of some of the mycotoxins as has been previously reported (Okeke et al., 2018).

In summary, our study indicates that the processing of *kunu* is mediated by several core bacterial phylotypes dominated by members of the LAB, although acetic acid bacteria and other bacteria with unknown functional roles constitute the core bacterial community. We have also further established that *kunu* could be a safe beverage for consumption in terms of mycotoxin contents depending on the grain inputs. Thus, we propose the combination of millet, white sorghum, cloves, ginger and sweet potato, which are usually minimally contaminated by mycotoxins, as possible combination of grains and additives for *kunu* production. This proposal will help to minimize mycotoxin exposure in consumers. In addition, the aforementioned ingredients could be supplemented with some underutilized crops (e.g., Bambara nut, coconut, finger millet (*acha*) and sesame seed) that may be less prone to mycotoxins. The extract from tiger nut may be more useful compared to the whole tiger nut; this will further reduce mycotoxin exposure while enhancing nutritional content and imparting additional flavor. Furthermore, it is imperative to source high quality grains and apply simple first-line grain processing interventions (e.g., sorting and floatation washing) to keep mycotoxin levels in the starting materials at the barest minimum in order to encourage a further mycotoxin reduction to non-detectable/safe limits in the final *kunu* product. There is a need to explore the core phylotypes reported in this study by isolating and ascertaining their probiotic properties and suitability as starters for up-scaling of some of the cereal-based traditional fermented foods. This study has further laid the foundation for the potential discovery of novel mycotoxin detoxifiers from *kunu*. Overall, the data provided herein are highly relevant to experts in the food microbiology and safety, food processing and technology, microbial ecology and molecular biology horizons.

## Author Contributions

CNE conceived the study. CNE, MS, OO, RA, CN, and RK designed the study. IC-O, KA, OO, OA, OE, CNE, and MS performed the experiments in Nigeria, South Africa, and Austria. MS, OE, DvW, CNE, and KA analyzed the data. CNE, CN, RA, JH, CTE, and RK supervised the overall study. KA, OE, DvW, and CNE drafted the manuscript. All authors reviewed and approved the manuscript.

## Conflict of Interest Statement

The authors declare that the research was conducted in the absence of any commercial or financial relationships that could be construed as a potential conflict of interest.

## Footnotes

1 https://www.ncbi.nlm.nih.gov/sra
